# Association between fine particulate matter and eczema: A cross-sectional study of the *All of Us* Research Program and the Center for Air, Climate, and Energy Solutions

**DOI:** 10.1371/journal.pone.0310498

**Published:** 2024-11-13

**Authors:** Gloria F. Chen, Erica Hwang, Charles E. Leonard, Jeffrey M. Cohen

**Affiliations:** 1 Yale School of Medicine, New Haven, Connecticut, United States of America; 2 Center for Real-World Effectiveness and Safety of Therapeutics, Perelman School of Medicine, University of Pennsylvania, Philadelphia, Pennsylvania, United States of America; 3 Department of Biostatistics, Epidemiology, and Informatics, Perelman School of Medicine, University of Pennsylvania, Philadelphia, Pennsylvania, United States of America; 4 Leonard Davis Institute of Health Economics, University of Pennsylvania, Philadelphia, Pennsylvania, United States of America; 5 Department of Dermatology, Yale School of Medicine, New Haven, Connecticut, United States of America; 6 Department of Biomedical Informatics and Data Science, Yale School of Medicine, New Haven, Connecticut, United States of America; Kyung Hee University School of Medicine, REPUBLIC OF KOREA

## Abstract

**Background:**

The prevalence of eczema has increased with industrialization. Industrial practices generate ambient air pollution, including fine particulate matter of diameter ≤ 2.5μm (PM_**2.5**_). Studies investigating the relationship between PM_**2.5**_ and eczema in the US are scarce. The objective of this study was to determine the risk of eczema with PM_**2.5**_ exposure in a diverse national cohort of American adults.

**Methods:**

In this cross-sectional study, eczema cases in the *All of Us* Research Program were linked via three-digit zip code to average annual PM_2.5_ concentrations from the Center for Air, Climate, and Energy Solutions. Eczema cases and controls were compared using Pearson’s χ^2^ test for categorical variables and one-way analysis of variance for continuous variables. The relationship between PM_2.5_ and eczema was assessed via logistic regression adjusting for demographic factors, smoking, and atopic comorbidities.

**Results:**

Individuals with eczema (n = 12,695) lived in areas with significantly higher PM_2.5_ concentrations than did individuals without eczema (n = 274,127) (0.83 x 10 μg/m^3^ v. 0.81 x 10 μg/m^3^, *P* < .001). PM_2.5_ concentration was significantly associated with eczema in univariable analysis (odds ratio 1.97, 95% confidence interval 1.77–2.19, *P* < .001), and in multivariable analyses, both controlling for demographics and smoking status (odds ratio 2.21, 95% confidence interval 1.98–2.47, *P* < .001) and with the addition of atopic comorbidities (odds ratio 2.38, 95% confidence interval 2.12–2.67, *P* < .001).

**Conclusions:**

The odds of eczema increased with greater PM_2.5_ concentration in this large, diverse, adult American cohort. Ambient air pollution is an environmental hazard that influences inflammatory skin disease, suggesting possible targeted interventions.

## Introduction

Prevalence of eczema is difficult to characterize given variable case definitions, sampling methods, and time period of study. Epidemiologic studies of US-based adult cohorts have found prevalences ranging from 5.5% to 10.1%, and a study of a pediatric cohort found a 12-month prevalence of 10.7% [1–4]. Regardless, the prevalence of eczema has increased globally with industrialization, suggesting a possible contribution from environmental factors [5]. One ubiquitous environmental exposure is ambient air pollution (AAP). Exposure to AAP has deleterious health effects and in 2019, contributed to 4.2 million deaths worldwide [6]. Fine particulate matter, referring to particles with aerodynamic diameter measuring less than 2.5 μm (PM_2.5_), constitutes a major component AAP and may be associated with eczema [7]. Particles of this size are small enough to travel deep into the airways and potentially diffuse across alveolar epithelial cells, enter cells themselves, and spread through the vasculature or lymph to reach other organs [8, 9]. Given that such small particles can reach the distal airways and enter the body through alveoli, these particles may also diffuse across and enter cells of the skin. Indeed, an *in vitro* model provides evidence of infiltration into the stratum corneum by PM_2.5_ after incubation of reconstructed human epidermis with 100 μg/mL of PM_2.5_ [10]. PM_2.5_ has a myriad of different components, a major portion of which are polycyclic aromatic hydrocarbons (PAH) which readily diffuse through the stratum corneum [11, 12]. PM_2.5_ as a whole may contribute to the development or exacerbation of eczema by inducing skin barrier dysfunction, oxidative damage, and inflammatory dysfunction via modulation of the aryl hydrocarbon receptor (AhR) pathway [7]. Furthermore, pre-existing barrier dysfunction due to eczema likely facilitates cutaneous penetration by PM_2.5_ [13].

Studies investigating the impact of PM_2.5_ on eczema in the US are scarce. The 2007–2008 National Survey of Children’s Health found that higher annual PM_2.5_ averaged by US state was associated with lower eczema prevalence, but positively associated with greater severity of eczema during cold months [14]. In a cohort composed primarily of adults living in North Carolina, PM_2.5_ was positively associated with self-reported eczema or psoriasis diagnoses, but only in mixture with other pollutants [15]. Following the 2018 California Camp Fire in San Francisco, dermatology clinic visits for eczema in children and adults increased, as well as visits for eczema and itch in older adults relative to younger adults [16, 17]. These investigations present a mixed picture of the potential relationship between PM_2.5_ and eczema across the US, with conflicting national results alongside strong local findings. This study seeks to determine the risk of eczema with PM_2.5_ exposure in a diverse national cohort of American adults.

## Methods

In this cross-sectional study, eczema cases were identified via electronic health records (EHR) in the *All of Us* Research Program (AoURP), a National Institutes of Health program that prioritizes recruitment of participants from demographic groups underrepresented in biomedical research, including racial, ethnic, sexual, and gender minorities. AoURP obtained informed consent in written form from all participants. All data were fully anonymized before researcher access. Analysis in this study was conducted with AoURP Controlled Tier Dataset v7 (C2022Q4R11). Participants without available EHR data in AoURP were excluded. Eczema and comorbidities including asthma, allergic rhinitis, food allergy, and eosinophilic esophagitis cases were identified via EHR. Demographic data, including date of birth, sex at birth, race, ethnicity, and annual household income, were obtained from survey data, as was information on smoking status. Age was calculated for each participant based on the C2022Q4R11 data cutoff date of July 1, 2022. Body mass index (BMI) data were calculated based on height and weight measurements taken at enrollment. To protect participant privacy and reduce the risk of re-identification, the most granular geolocation data available in AoURP is three-digit zip code, based on participants’ self-reported addresses. Participants without zip code data available were excluded from this study.

Local air pollution data was obtained from the Center for Air, Climate, and Energy Solutions (CACES) which provides average annual pollutant concentrations in census tracts based on data from EPA monitors, satellites, and land use [18, 19]. Since AoURP geolocation is given as partial zip codes only, CACES census tract geolocation data were linked to zip codes via United States Department of Housing and Urban Development’s (HUD’s) Zip Code Crosswalk files, also termed HUD-United States Postal Service (USPS) Crosswalk files [20]. Zip code-to-tract HUD-USPS Crosswalk data was selected for the 2nd quarter of calendar year 2022, based on proximity to the July 1, 2022 AoURP data cutoff date. PM_2.5_ concentration for each zip code was calculated by weighted average of census tract PM_2.5_ concentrations, where weights corresponded to ratio of residential addresses in a census tract within a given zip code to total residential addresses in that zip code. Three-digit zip code-level data were then calculated by taking the median of PM_2.5_ concentrations in corresponding five-digit zip codes. Median was selected as the measure of central tendency most robust to extreme outliers. PM_2.5_ concentrations were standardized from units of μg/m^3^ to units of 10 μg/m^3^ to facilitate interpretation and comparison with other studies.

Urbanity was drawn from the U.S. Census Bureau’s block-level urban-rural classifications for the 2020 Census [21]. Binary values were assigned for each census block with urban = 1 and rural = 0, and average census tract urbanity was calculated as mean block urbanity. As with PM_2.5_ concentrations, census tracts were linked to five-digit zip codes by zip-code-to-tract HUD-USPS Crosswalk 2nd quarter 2022 data, and urbanity for each zip code was calculated as average census tract urbanity weighted by ratio of residential addresses. Three-digit zip code urbanity was represented by median five-digit zip code urbanity.

AoURP participants diagnosed with eczema were compared with a control group of participants without eczema diagnoses using Pearson’s χ^2^ test for categorical variables and one-way analysis of variance for continuous variables using package “tableone,” version 0.13.2. Multicollinearity was tested by generalized variance inflation factor with a test threshold = 2 for highly correlated variables, using package “car,” version 3.1.2. The relationship between PM_2.5_ and eczema was assessed via logistic regression using package “stats,” version 4.3.1. Three logistic models were analyzed:

1. A univariable model with only PM_2.5_ concentration as an independent variable and eczema as dependent variable,
2. A multivariable model with PM_2.5_ concentration as an independent variable; age, sex, race/ethnicity, income, urbanity, BMI, and smoking included as covariates; and eczema as dependent variable, and
3. A multivariable model with PM_2.5_ concentration as an independent variable; age, sex, race/ethnicity, income, urbanity, BMI, smoking, food allergy, allergic rhinitis, asthma, and eosinophilic esophagitis as covariates; and eczema as dependent variable.

PM_2.5_ concentration in the year 2015 was the primary focus of the analysis as the most recent year of CACES data available. Two-sided alpha = .05 was considered significant.

## Results

**Fig 1:**
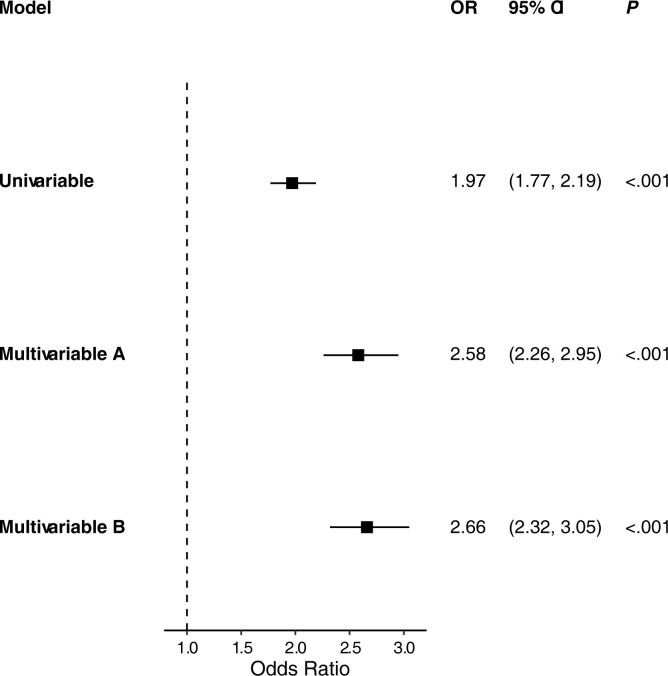
Univariable and multivariable associations of PM_2.5_ with eczema. PM_2.5_ –particulate matter with diameter ≤ 2.5 μm; OR–odds ratio; CI–confidence interval. ^A^Adjusted for age, sex, race/ethnicity, income, urbanity, body mass index (BMI), and smoking. ^B^Adjusted for age, sex, race/ethnicity, BMI, income, smoking, food allergy, allergic rhinitis, asthma, and eosinophilic esophagitis. Odds ratios are calculated for PM_2.5_ concentrations in units of 10 μg/m^3^.

Among 287,011 participants in AoURP Controlled Tier with demographic, smoking, and EHR data available, 286,826 (99.9%) participants had zip code data from 788 unique three-digit zip codes available for linkage to CACES PM_2.5_ data. 286,766 (99.9%) of these participants were successfully crosslinked via HUD’s Zip Code Crosswalk data, and 60 participants were unable to be crosslinked due to location in non-contiguous regions of the US without available PM_2.5_ data. 12,695 participants were diagnosed with eczema (mean age, 58.45; standard deviation 16.80), and 274,127 were not diagnosed with eczema (mean age, 54.85; standard deviation 16.95). Individuals with eczema lived in areas with significantly higher PM_2.5_ concentrations than did individuals without eczema (0.83 x 10 μg/m^3^ vs. 0.81 x 10 μg/m^3^, *P* < .001). (**Table 1**). Multicollinearity was not detected with all generalized variance inflation factor tests under threshold = 2 (**S1 Table**). PM_2.5_ concentration was significantly associated with eczema in univariable analysis (odds ratio [OR] 1.97, 95% confidence interval [CI] 1.77–2.19, *P* < .001; Akaike information criterion [AIC] 103823), as well as in multivariable analysis adjusting for age, sex, race/ethnicity, urbanity, BMI, income, and smoking status (OR 2.58, 95% CI 2.26–2.95, *P* < .001; AIC 95421) and with the addition of atopic comorbidities including food allergy, allergic rhinitis, asthma, and eosinophilic esophagitis (OR 2.66, 95% CI 2.32–3.05, *P* < .001; AIC 89968) (**Fig 1**).

**Table 1:** Clinical characteristics of eczema cases and controls.

	Eczema Cases^a^		Controls		*P*
**n**	12699		274127		
**Age, mean (SD)**	58.44	(16.80)	54.85	(16.95)	< .001
**Sex (%)**					< .001
** Female**	8351	(65.8)	163951	(59.8)	
** Male**	4077	(32.1)	104579	(38.1)	
** Intersex**	<20		56	(0.0)	
** Unknown**	270	(2.1)	5541	(2.0)	
**Race/Ethnicity (%)**					< .001
** Asian**	501	(3.9)	9299	(3.4)	
** Black**	2177	(17.1)	56591	(20.6)	
** Hispanic**	1699	(13.4)	51570	(18.8)	
** MENA**	100	(0.8)	2777	(1.0)	
** NHPI**	22	(0.2)	742	(0.3)	
** White**	7686	(60.5)	142643	(52.0)	
** Unknown**	513	(4.0)	10505	(3.8)	
**Income (%)**					< .001
** Less than $10,000**	1304	(10.3)	39509	(14.4)	
** $10,000 to $25,000**	1367	(10.8)	32582	(11.9)	
** $25,000 to $35,000**	867	(6.8)	19390	(7.1)	
** $35,000 to $50,000**	1091	(8.6)	21148	(7.7)	
** $50,000 to $75,000**	1475	(11.6)	27703	(10.1)	
** $75,000 to $100,000**	1246	(9.8)	21105	(7.7)	
** $100,000 to $150,000**	1526	(12.0)	25671	(9.4)	
** $150,000 to $200,000**	687	(5.4)	11803	(4.3)	
** More than $200,000**	950	(7.5)	16555	(6.0)	
** Unknown**	2186	(17.2)	58661	(21.4)	
**Urbanity, mean (SD)**	0.49	(0.29)	0.51	(0.28)	< .001
**BMI, mean (SD)**	30.24	(7.59)	29.88	(7.64)	< .001
**Ever smoker (%)**					.006
** Yes**	4906	(38.6)	109594	(40.0)	
** No**	7394	(58.2)	156542	(57.1)	
** Unknown**	399	(3.1)	7991	(2.9)	
**Food allergy**^**b**^ **(%)**	113	(0.9)	432	(0.2)	< .001
**Allergic rhinitis**^**c**^ **(%)**	5759	(45.4)	42576	(15.5)	< .001
**Asthma**^**d**^ **(%)**	4226	(33.3)	42966	(15.7)	< .001
**Eosinophilic esophagitis**^**e**^ **(%)**	95	(0.7)	689	(0.3)	< .001
**PM**_**2.5**_ **concentration, mean (SD)**	0.83	(0.16)	0.81	(0.17)	< .001

SD–standard deviation; MENA–Middle Eastern or North African; NHPI–Native Hawaiian or other Pacific Islander; BMI–body mass index; PM_2.5_ –particulate matter with diameter ≤ 2.5 μm. ^a^SNOMED 43116000; ICD-10-CM L20, L30.0, L30.1; ICD-9-CM 691, 705.81; excluding SNOMED 88996004; ICD-9-CM 697, 698.3. ^b^SNOMED 419452009, ICD-10-CM T78.0, ICD-9-CM 995.6. ^c^SNOMED 61582004; ICD-10-CM J30.1, J30.2, J30.5, J30.8, J30.9; ICD-9-CM 477. ^d^SNOMED 195967001; ICD-10-CM J45; ICD-9-CM 493. ^e^SNOMED 235599003; ICD-10-CM K20.0; ICD-9-CM 530.13. Values less than 20 are noted as <20 to protect the privacy of participants per the *All of Us* Research Program data and statistics dissemination policy. PM_2.5_ concentrations are presented in units of 10 μg/m^3^.

## Discussion

Eczema was positively associated with PM_2.5_ concentration in this large, diverse, adult American cohort. Several global studies of PM_2.5_ exposure and risk of eczema report similar results. An analysis of ground-level pollution data in Taiwan found an adjusted OR of 1.63 (95% CI 1.22–2.16, *P* < .005) for physician-diagnosed eczema in adults for a 10 μg/m^3^ increase in PM_2.5_ concentration [22]. In a cohort of German women 55 years of age and older, the OR of incident eczema with a 10 μg/m^3^ increase in PM_2.5_ was 2.20 (95% CI 1.13–4.32, *P* < .05) [23]. Finally, an Australian study found that satellite-based estimates of ground level PM_2.5_ were positively associated with positive skin prick test as a proxy for atopic eczema, with OR 2.40 (95% CI 1.20–5.15, *P* = .017) for every 10 μg/m^3^ increase in PM_2.5_ [24]. Together with this study, findings from countries across the world support an approximately two-fold increased risk of eczema with 10 μg/m^3^ increases in PM_2.5_.

Strengths of this study include EHR-validated diagnoses, ground-level PM_2.5_ concentrations from 788 distinct locations, and a nationwide cohort with representation from marginalized communities most impacted by AAP. Limitations include lack of clinical detail such as text from visit notes, geolocation resolution of three-digit ZIP code, and PM_2.5_ data availability only to 2015. Furthermore, while AoURP aims to reflect the diversity present in the United States, it is not designed to reflect the representative demographics of the United States as a whole nor is it designed to sample all regions of the country proportionately. Other epidemiological studies have shown that eczema is less prevalent in rural settings, with the lowest prevalence in the United States seen in the Midwest region; eczema may also be associated with latitude [25–27]. Future studies aiming to explore regional differences in the relationship between AAP and eczema can mitigate frame error through probability-based sampling of a database that guarantees regional coverage, standardization to factors of interest, and stratified analyses. Interestingly, OR for risk conferred by elevated PM_2.5_ concentration increases with addition of covariates. This phenomenon may be attributed to suppressor variables also correlated with AAP in the US that increase predictive power of PM_2.5_, including race/ethnicity, income, and atopic disease [28, 29]. Relative to the univariable model and the multivariable model without atopic comorbidities, the multivariable model B, which included all covariates of age, sex, race/ethnicity, income, urbanity, BMI, smoking, food allergy, allergic rhinitis, asthma, and eosinophilic esophagitis, had the lowest AIC indicating that it was the best fit model in this study.

While cross-sectional analysis does not convey directionality, individual eczema diagnoses leading to widespread increases in local PM_2.5_ levels would be difficult to imagine. More likely, increased PM_2.5_ exposure would influence the risk of eczema, potentially through modulation of the aryl hydrocarbon receptor (AhR) pathway and generation of oxidative stress, leading to impairment in the epidermal barrier and associated inflammation [7]. PAHs are produced by carbon fuel combustion, and as a component of PM_2.5_, can diffuse through the stratum corneum where they serve as ligands for AhR [7, 11, 12, 30, 31]. Activation of AhR in keratinocytes has been shown in mice to induce an eczema phenotype with severe, pruritic skin lesions as well as a T helper 2 (Th2)-mediated immune response [32]. PAHs increase the gene expression and levels of artemin, a mediator of AhR in epidermal cells which induces epidermal hyperinnervation and pruritus hypersensitivity [31, 33]. Further, AhR mediates production of reactive oxygen species (ROS) by increasing cytochrome P450 expression, potentially leading to air pollution-induced oxidative damage and inflammation [34]. Both PM_2.5_-exposed skin cells and skin of patients with eczema exhibit oxidative damage [35, 36]. Individuals with eczema may be at increased risk for transcutaneous PM_2.5_ absorption and subsequent activation of the AhR pathway given that penetration of PM_2.5_ into skin tissue is enhanced by disruption of the skin barrier [13, 37].

Our finding of an approximately two-fold increased risk of eczema per 10 μg/m^3^ rise in PM_2.5_ is clinically relevant as well as actionable, as the United States Environmental Protection Agency’s Air Quality Index (AQI) is based on differences in PM_2.5_ concentrations of comparable orders of magnitude [38]. Individuals with eczema may be at elevated risk for disease exacerbation or acute flares when AQI reaches the “moderate” category (12.1–35.4 μg/m^3^) compared to the “good” category (0–12.0 μg/m^3^), with risk increasing at even higher AQI. When AQI reaches levels of “moderate” or worse, patients may be advised to stay indoors, filter indoor air, or cover exposed skin outdoors. Additionally, since evidence suggests that PM_2.5_ may induce eczema via non-canonical AhR signaling, activation of canonical AhR signaling may balance such immune dysregulation; the AhR agonist tapinarof is currently under investigation for treatment of eczema and may be of particular utility for those exposed to elevated PM_2.5_ levels [39, 40]. Further understanding of the relationship between AAP and eczema will serve to refine these recommendations.

## Supporting information

S1 Table Generalized variance inflation factor tests. (DOCX)

S1 File Univariable and multivariable regression results. (DOCX)
