# Supplementary material for: Association between fine particulate matter and eczema: A cross-sectional study of the All of Us Research Program and the Center for Air, Climate, and Energy Solutions
Source: PLoS One. 2024 Nov 13;19(11):e0310498. doi: 10.1371/journal.pone.0310498 (PMC11560005; doi:10.1371/journal.pone.0310498)
Supplement: S1 Table — (DOCX) [file pone.0310498.s001.docx]

**S1 Table. Generalized Variance Inflation Factor tests**

Test threshold for highly correlated variables:

$${GVIF}^{\frac{1}{2*df}}\geq2$$

where

$$GVIF=Generalized Variance Inflation Factor$$

and

$$df=degrees of freedom$$

vif(glm_multi)

| A matrix: 12 × 3 of type dbl | | | |
| --- | --- | --- | --- |
|  | **GVIF** | **Df** | **GVIF^(1/(2*Df))** |
| **age** | 1.115938 | 1 | 1.056380 |
| **sex** | 1.242239 | 3 | 1.036814 |
| **race_assign** | 1.770537 | 6 | 1.048758 |
| **income** | 1.420696 | 9 | 1.019700 |
| **smok100U** | 1.080621 | 1 | 1.039529 |
| **bmi** | 1.086783 | 1 | 1.042489 |
| **asthma** | 1.161311 | 1 | 1.077641 |
| **allerg_rhin** | 1.147610 | 1 | 1.071266 |
| **food_allergy** | 1.012197 | 1 | 1.006080 |
| **eoe** | 1.006339 | 1 | 1.003164 |
| **med_urban** | 1.474877 | 1 | 1.214445 |
| **pm2510_2015** | 1.427222 | 1 | 1.194664 |
