# Supplementary material for: Association between fine particulate matter and eczema: A cross-sectional study of the All of Us Research Program and the Center for Air, Climate, and Energy Solutions
Source: PLoS One. 2024 Nov 13;19(11):e0310498. doi: 10.1371/journal.pone.0310498 (PMC11560005; doi:10.1371/journal.pone.0310498)
Supplement: S1 File — (DOCX) [file pone.0310498.s002.docx]

**Univariable**

glm_uni <- glm(eczema~(pm2510_2015),family="binomial", data = air_ehr_df)

summary(glm_uni)

exp(coef(glm_uni))

exp(confint(glm_uni))

Call:

glm(formula = eczema ~ (pm2510_2015), family = "binomial", data = air_ehr_df)

Deviance Residuals:

Min 1Q Median 3Q Max

-0.3730 -0.3121 -0.3000 -0.2868 2.7020

Coefficients:

Estimate Std. Error z value Pr(>|z|)

(Intercept) -3.62420 0.04632 -78.24 <2e-16 ***

pm2510_2015 0.67592 0.05504 12.28 <2e-16 ***

---

Signif. codes: 0 ‘***’ 0.001 ‘**’ 0.01 ‘*’ 0.05 ‘.’ 0.1 ‘ ’ 1

(Dispersion parameter for binomial family taken to be 1)

Null deviance: 103972 on 286765 degrees of freedom

Residual deviance: 103819 on 286764 degrees of freedom

AIC: 103823

Number of Fisher Scoring iterations: 6

**(Intercept)**

0.0266705165937378

**pm2510_2015**

1.96584846540706

Waiting for profiling to be done...

A matrix: 2 × 2 of type dbl

**2.5 % 97.5 %**

**(Intercept)** 0.02435028 0.02919837

**pm2510_2015** 1.76500585 2.19003748

**Multivariable Model A**

glm_multi <- glm(eczema~

age+sex+race_assign+income+smok100U

+bmi

+med_urban

+pm2510_2015,

family="binomial", data = urban_ad_air_df)

summary(glm_multi)

exp(coef(glm_multi)) #adjusted OR

exp(confint(glm_multi)) #confidence intervals

Call:

glm(formula = eczema ~ age + sex + race_assign + income + smok100U +

bmi + med_urban + pm2510_2015, family = "binomial", data = urban_ad_air_df)

Coefficients:

Estimate Std. Error z value Pr(>|z|)

(Intercept) -4.210244 0.087096 -48.340 < 2e-16 ***

age 0.012335 0.000605 20.389 < 2e-16 ***

sexintersex -0.827569 1.010662 -0.819 0.41288

sexmale -0.301909 0.020617 -14.644 < 2e-16 ***

sexunknown -0.023063 0.070859 -0.325 0.74482

race_assignBlack -0.295549 0.055068 -5.367 8.01e-08 ***

race_assignHispanic -0.366955 0.055503 -6.611 3.81e-11 ***

race_assignMENA -0.368264 0.114837 -3.207 0.00134 **

race_assignNHPI -0.500134 0.227371 -2.200 0.02783 *

race_assignUnknown -0.101861 0.070490 -1.445 0.14844

race_assignWhite -0.156339 0.050703 -3.083 0.00205 **

income10k_25k -0.254440 0.040955 -6.213 5.21e-10 ***

income150k_200k 0.019404 0.049437 0.392 0.69469

income25k_35k -0.184406 0.045761 -4.030 5.58e-05 ***

income35k_50k -0.081723 0.042759 -1.911 0.05597 .

income50k_75k -0.089622 0.039339 -2.278 0.02271 *

income75k_100k -0.009671 0.041263 -0.234 0.81469

incomeless_than_10k -0.369176 0.042784 -8.629 < 2e-16 ***

incomemore_than_200k 0.013219 0.044745 0.295 0.76767

incomeUnknown -0.351577 0.037533 -9.367 < 2e-16 ***

smok100UYes -0.029863 0.020085 -1.487 0.13707

bmi 0.009166 0.001242 7.377 1.62e-13 ***

med_urban -0.214149 0.039480 -5.424 5.82e-08 ***

pm2510_2015 0.947759 0.067818 13.975 < 2e-16 ***

---

Signif. codes: 0 ‘***’ 0.001 ‘**’ 0.01 ‘*’ 0.05 ‘.’ 0.1 ‘ ’ 1

(Dispersion parameter for binomial family taken to be 1)

Null deviance: 96856 on 266139 degrees of freedom

Residual deviance: 95373 on 266116 degrees of freedom

(20626 observations deleted due to missingness)

AIC: 95421

Number of Fisher Scoring iterations: 6

**(Intercept)**

0.0148427502503799

**age**

1.0124118032896

**sexintersex**

0.437110496915521

**sexmale**

0.73940533043163

**sexunknown**

0.977200654054932

**race_assignBlack**

0.744122695496549

**race_assignHispanic**

0.692840526276656

**race_assignMENA**

0.691934319156917

**race_assignNHPI**

0.606449605239148

**race_assignUnknown**

0.903155033305615

**race_assignWhite**

0.855268912123206

**income10k_25k**

0.775350678013513

**income150k_200k**

1.01959329740475

**income25k_35k**

0.831598477012615

**income35k_50k**

0.921527675447943

**income50k_75k**

0.914276510646306

**income75k_100k**

0.990375672372285

**incomeless_than_10k**

0.691303454575788

**incomemore_than_200k**

1.01330649621676

**incomeUnknown**

0.703577739615253

**smok100UYes**

0.970578530692698

**bmi**

1.00920832567749

**med_urban**

0.807227821280934

**pm2510_2015**

2.57992129915383

Waiting for profiling to be done...

| A matrix: 24 × 2 of type dbl | | |
| --- | --- | --- |
|  | **2.5 %** | **97.5 %** |
| **(Intercept)** | 0.01250616 | 0.01759555 |
| **age** | 1.01121325 | 1.01361433 |
| **sexintersex** | 0.02467517 | 1.99553942 |
| **sexmale** | 0.71006518 | 0.76983511 |
| **sexunknown** | 0.84868341 | 1.12051721 |
| **race_assignBlack** | 0.66861496 | 0.82974300 |
| **race_assignHispanic** | 0.62197888 | 0.77318903 |
| **race_assignMENA** | 0.54956211 | 0.86244603 |
| **race_assignNHPI** | 0.37674625 | 0.92275491 |
| **race_assignUnknown** | 0.78657317 | 1.03698536 |
| **race_assignWhite** | 0.77525738 | 0.94576192 |
| **income10k_25k** | 0.71551545 | 0.84013111 |
| **income150k_200k** | 0.92494357 | 1.12277263 |
| **income25k_35k** | 0.76002984 | 0.90937927 |
| **income35k_50k** | 0.84730742 | 1.00194101 |
| **income50k_75k** | 0.84642131 | 0.98756114 |
| **income75k_100k** | 0.91333854 | 1.07370678 |
| **incomeless_than_10k** | 0.63566252 | 0.75174056 |
| **incomemore_than_200k** | 0.92797709 | 1.10591531 |
| **incomeUnknown** | 0.65375065 | 0.75737755 |
| **smok100UYes** | 0.93307222 | 1.00950711 |
| **bmi** | 1.00674497 | 1.01166035 |
| **med_urban** | 0.74721684 | 0.87228569 |
| **pm2510_2015** | 2.25925742 | 2.94727246 |

**Multivariable Model B**

glm_multi <- glm(eczema~

age+sex+race_assign+income+smok100U

+bmi

+asthma+allerg_rhin+food_allergy+eoe

+med_urban

+pm2510_2015,

family="binomial", data = urban_ad_air_df)

summary(glm_multi)

exp(coef(glm_multi)) #adjusted OR

exp(confint(glm_multi)) #confidence intervals

Call:

glm(formula = eczema ~ age + sex + race_assign + income + smok100U +

bmi + asthma + allerg_rhin + food_allergy + eoe + med_urban +

pm2510_2015, family = "binomial", data = urban_ad_air_df)

Coefficients:

Estimate Std. Error z value Pr(>|z|)

(Intercept) -4.3254634 0.0891906 -48.497 < 2e-16 ***

age 0.0095382 0.0006182 15.430 < 2e-16 ***

sexintersex -0.6889352 1.0139109 -0.679 0.49683

sexmale -0.1401201 0.0210814 -6.647 3.00e-11 ***

sexunknown 0.0338818 0.0723825 0.468 0.63972

race_assignBlack -0.3489829 0.0558167 -6.252 4.04e-10 ***

race_assignHispanic -0.3835673 0.0563109 -6.812 9.65e-12 ***

race_assignMENA -0.4227923 0.1163708 -3.633 0.00028 ***

race_assignNHPI -0.5416760 0.2301300 -2.354 0.01858 *

race_assignUnknown -0.1943043 0.0718363 -2.705 0.00683 **

race_assignWhite -0.2408843 0.0514502 -4.682 2.84e-06 ***

income10k_25k -0.2760272 0.0416085 -6.634 3.27e-11 ***

income150k_200k 0.0211990 0.0503092 0.421 0.67348

income25k_35k -0.1839543 0.0464961 -3.956 7.61e-05 ***

income35k_50k -0.0844725 0.0434913 -1.942 0.05210 .

income50k_75k -0.1021105 0.0400180 -2.552 0.01072 *

income75k_100k -0.0141198 0.0419860 -0.336 0.73665

incomeless_than_10k -0.3640530 0.0432755 -8.412 < 2e-16 ***

incomemore_than_200k 0.0249952 0.0455513 0.549 0.58319

incomeUnknown -0.3228027 0.0381096 -8.470 < 2e-16 ***

smok100UYes -0.0023353 0.0203841 -0.115 0.90879

bmi -0.0002778 0.0012957 -0.214 0.83021

asthma 0.5999775 0.0222047 27.020 < 2e-16 ***

allerg_rhin 1.2226882 0.0207489 58.928 < 2e-16 ***

food_allergy 0.9142278 0.1137719 8.036 9.31e-16 ***

eoe 0.5195012 0.1189399 4.368 1.26e-05 ***

med_urban -0.1648839 0.0405594 -4.065 4.80e-05 ***

pm2510_2015 0.9780544 0.0698150 14.009 < 2e-16 ***

---

Signif. codes: 0 ‘***’ 0.001 ‘**’ 0.01 ‘*’ 0.05 ‘.’ 0.1 ‘ ’ 1

(Dispersion parameter for binomial family taken to be 1)

Null deviance: 96856 on 266139 degrees of freedom

Residual deviance: 89912 on 266112 degrees of freedom

(20626 observations deleted due to missingness)

AIC: 89968

Number of Fisher Scoring iterations: 6

**(Intercept)**

0.0132274197408097

**age**

1.00958382818172

**sexintersex**

0.50211042630898

**sexmale**

0.869253802654194

**sexunknown**

1.03446235950876

**race_assignBlack**

0.705405172923036

**race_assignHispanic**

0.681426217373896

**race_assignMENA**

0.655214731978044

**race_assignNHPI**

0.58177236639097

**race_assignUnknown**

0.823407335764939

**race_assignWhite**

0.785932582047871

**income10k_25k**

0.758792314518056

**income150k_200k**

1.02142526701939

**income25k_35k**

0.831973834870839

**income35k_50k**

0.918996935561875

**income50k_75k**

0.902929731230888

**income75k_100k**

0.985979377654215

**incomeless_than_10k**

0.694854366153591

**incomemore_than_200k**

1.02531018484871

**incomeUnknown**

0.724116703136852

**smok100UYes**

0.997667459006743

**bmi**

0.999722204316658

**asthma**

1.82207784708249

**allerg_rhin**

3.39630529672639

**food_allergy**

2.4948480192526

**eoe**

1.68118880256969

**med_urban**

0.847992127864228

**pm2510_2015**

2.65927729322584

Waiting for profiling to be done...

| A matrix: 28 × 2 of type dbl | | |
| --- | --- | --- |
|  | **2.5 %** | **97.5 %** |
| **(Intercept)** | 0.01109943 | 0.01574512 |
| **age** | 1.00836258 | 1.01080900 |
| **sexintersex** | 0.02825417 | 2.31645654 |
| **sexmale** | 0.83400788 | 0.90585846 |
| **sexunknown** | 0.89579553 | 1.18980374 |
| **race_assignBlack** | 0.63288247 | 0.78770804 |
| **race_assignHispanic** | 0.61075134 | 0.76163840 |
| **race_assignMENA** | 0.51889649 | 0.81922468 |
| **race_assignNHPI** | 0.35968032 | 0.89051515 |
| **race_assignUnknown** | 0.71522107 | 0.94790587 |
| **race_assignWhite** | 0.71134137 | 0.87033540 |
| **income10k_25k** | 0.69933866 | 0.82324402 |
| **income150k_200k** | 0.92503759 | 1.12673107 |
| **income25k_35k** | 0.75928430 | 0.91110845 |
| **income35k_50k** | 0.84377182 | 1.00062934 |
| **income50k_75k** | 0.83480443 | 0.97660352 |
| **income75k_100k** | 0.90799882 | 1.07045964 |
| **incomeless_than_10k** | 0.63831388 | 0.75633306 |
| **incomemore_than_200k** | 0.93749540 | 1.12079398 |
| **incomeUnknown** | 0.67207275 | 0.78036740 |
| **smok100UYes** | 0.95855487 | 1.03829282 |
| **bmi** | 0.99717839 | 1.00225601 |
| **asthma** | 1.74435579 | 1.90299369 |
| **allerg_rhin** | 3.26087834 | 3.53719006 |
| **food_allergy** | 1.98760588 | 3.10601728 |
| **eoe** | 1.32337183 | 2.11057113 |
| **med_urban** | 0.78328983 | 0.91827476 |
| **pm2510_2015** | 2.31968879 | 3.04989105 |
